# Collective excitations on a surface of topological insulator

**DOI:** 10.1186/1556-276X-7-163

**Published:** 2012-02-29

**Authors:** Dmitry K Efimkin, Yurii E Lozovik, Alexey A Sokolik

**Affiliations:** 1 Institute for Spectroscopy, Russian Academy of Sciences, Fizicheskaya 5, 142190, Troitsk, Moscow Region, Russia; 2 Moscow Institute of Physics and Technology, Institutskii Per. 9, 141700, Dolgoprudny, Moscow Region, Russia

## Abstract

We study collective excitations in a helical electron liquid on a surface of three-dimensional topological insulator. Electron in helical liquid obeys Dirac-like equation for massless particles and direction of its spin is strictly determined by its momentum. Due to this spin-momentum locking, collective excitations in the system manifest themselves as coupled charge- and spin-density waves. We develop quantum field-theoretical description of spin-plasmons in helical liquid and study their properties and internal structure. Value of spin polarization arising in the system with excited spin-plasmons is calculated. We also consider the scattering of spin-plasmons on magnetic and nonmagnetic impurities and external potentials, and show that the scattering occurs mainly into two side lobes. Analogies with Dirac electron gas in graphene are discussed.

**PACS**: 73.20.Mf; 73.22.Lp; 75.25.Dk.

## 1 Introduction

Topological insulator is a new class of solids with nontrivial topology, intrinsic to its band structure. Theoretical and experimental studies of topological insulators grow very rapidly in recent years (see [1,2] and references therein). Three-dimensional topological insulators are insulating in the bulk, but have gapless surface states with numerous unusual properties. These states are topologically protected against time-reversal invariant disorder. When gap is opened in surface states by time-reversal or gauge symmetry breaking, a spectacular magnetoelectric effect arises [3,4].

Recently a "new generation" of 3D topological insulators (the binary compounds Bi_2_Se_3_, Bi_2_Te_3 _and other materials), retaining topologically protected behavior at room temperatures, were predicted and studied experimentally [5-7]. Band structure of the surface states of these materials contains a single Dirac cone, where electrons obey 2D Dirac equation for massless particles. Direction of electron spin in these states is strictly determined by their momentum, so these states can be called as "helical" ones. Surface of topological insulator can be chemically doped, forming charged helical liquid. The spin-momentum locking leads to interesting transport phenomena including coupled diffusion of spin and charge [8], inverse galvano-magnetic effect (generation of spin polarization by electric current) [9] and giant spin rotation on an interface between normal metal and topological insulator [10]. The spin-momentum locking offers numerous opportunities for various spintronic applications.

Collective excitations (plasmons) in helical liquid on the surface of topological insulator was considered in [11]. It was shown that due to spin-momentum locking responses of charge and spin densities to external electromagnetic field are coupled to each other. Therefore the plasmons in the system should manifest themselves as coupled charge- and spin-density waves and can be called "spin-plasmons". In [12] application of spin-plasmons in spin accumulator device was proposed. Also surface plasmon-polaritons under conditions of topological magnetoelectric effect were considered in [13].

Properties of the states on a surface of 3D topological insulator are similar to those of electrons in graphene. Graphene is unique 2D carbon material with extraordinary electronic properties [14-16]. Its band structure contains two Dirac cones with electrons behaving as massless Dirac particles in their vicinities. Graphene is a perspective material for nanoelectronics due to large carrier mobilities at room temperature. Electronic interactions and collective excitations in graphene have been extensively studied (see [17] and references therein). In particular, the properties of plasmons [18,19] and hybrid plasmon-photon [20] and plasmon-phonon [21,22] modes were investigated theoretically and experimentally. It was realized recently that graphene is a fertile ground for quantum plasmonics [23] due to very small plasmon damping.

In this article, we develop quantum field-theoretical formalism to describe plasmons in graphene and spin-plasmons on a surface of 3D topological insulator based on random phase approximation (RPA). Problems of excitation, manipulation, scattering and detection of single plasmons can be conveniently considered using this approach. Thus, this approach can be especially useful for problems of plasmon quantum optics and quantum plasmonics. We use our approach here to study internal structure and properties of spin-plasmons in a helical liquid.

The rest of this article is organized as follows. In Section 2, we present a brief description of electronic states on a surface of topological insulator and in graphene. Next we develop the original quantum field-theoretical description of plasmons in Dirac electron gas in Section 3 and apply it further to study their properties. We consider internal structure of plasmons in Section 4 and important consequences of spin-momentum locking in Section 5. Scattering of plasmons on impurities and external potentials is considered in Section 6, and Section 7 is devoted to conclusions.

## 2 Dirac electrons

The low-energy effective Hamiltonians for electrons in helical liquid [24] and graphene [16] are

(1) $$\text{helical}\;\text{liquid:}\;\;H_{0}=v_{\text{F}}\left(p_{x}\sigma_{y}-p_{y}\sigma_{x}\right),$$

(2) $$\text{graphene:}\;\;H_{0}=v_{\text{F}}\left(p_{x}\sigma_{x}+p_{y}\sigma_{y}\right),$$

where the Fermi velocities of electrons *v*_F _are 6.2 × 10^5 ^m/s for the topological insulator Bi_2_Se_3 _and 10^6^m/s for graphene; the Pauli matrices *σ*_*x *_and *σ*_*y *_act in the spaces of electron spin projections (helical liquid) or sublattices (graphene). The eigenfunctions of (1)-(2) can be written as $e^{ip\cdot r}|f_{p\gamma }\rangle /\sqrt{S}$, where *S *is the system area and |*f*_**p***γ*_) is the spinor part of the eigenfunction, corresponding to electron with momentum **p **from conduction (*γ *= +1) or valence (*γ *= -1) band:

(3) $$\text{helical}\;\text{liquid:}\;\;|f_{p\gamma }\rangle =\frac{1}{\sqrt{2}}\left(\begin{array}{c} e^{-i\varphi_{p}/2}\\ \mathrm{i\gamma}e^{i\varphi_{p}/2} \end{array}\right),$$

(4) $$\text{graphene:}\;\;|f_{p\gamma }\rangle =\frac{1}{\sqrt{2}}\left(\begin{array}{c} e^{-i\varphi_{p}/2}\\ \gamma e^{i\varphi_{\text{p}}/2} \end{array}\right),$$

where *φ*_**p **_is a polar angle of the vector **p **(here and below we assume *ħ *= 1). Another difference between helical liquid on a surface of topological insulator and electron liquid in graphene is additional fourfold degeneracy *g *= 4 of electronic states in graphene by two spin projections and two nonequivalent valleys.

The value of electron spin in helical liquid and of pseudospin in graphene in the state |*f*_**p***γ*_〉 is

(5) $$\text{helical}\;\text{liquid:}\;\;\left\langle f_{p\gamma }\left|\sigma \right|f_{p\gamma }\right\rangle =\gamma \left[\hat{z}\times \hat{p}\right],$$

(6) $$\text{graphene:}\;\;\left\langle f_{p\gamma }\left|\sigma \right|f_{p\gamma }\right\rangle =\gamma \hat{p},$$

where $\hat{p}$ and $\hat{z}$ are unit vectors directed along the momentum **p **and the *z*-axis, respectively. We see that, in helical liquid, the spin of electron lies in the system plane and makes an angle 90° (in counterclockwise direction in the conduction band and inversely in the valence band) with its momentum. In graphene, the sublattice pseudospin of electron is directed along its momentum in conduction band and opposite to it in the valence band. Physically, a definite direction of the pseudospin in the system plane corresponds to definite phase shift between electron wave functions on different sublattices.

A starting point for quantum field-theoretical consideration of plasmons on the surface of topological insulator and in graphene is the many-body Hamiltonian of electrons with Coulomb interaction between them:

(7) $$\begin{array}{c} H=\underset{p\gamma }{\sum }\xi_{\mathrm{p\gamma}}a_{p\gamma }^{+}a_{p\gamma }+\frac{1}{2S}\underset{{\mathrm{qpp}}^{\prime }}{\sum }\underset{\gamma_{1}\gamma_{2}}{\sum }\underset{\gamma_{1}^{\prime }\gamma_{2}^{\prime }}{\sum }V_{q}\\ \;\;\;\;\times \left\langle f_{p+q,\gamma_{1}^{\prime }}|f_{p\gamma_{1}}\right\rangle \left\langle f_{p^{\prime }-q,\gamma_{2}^{\prime }}|f_{p^{\prime }\gamma_{2}}\right\rangle \\ \;\;\;\;\;\;\;\times a_{p+q,\gamma_{1}^{\prime }}^{+}a_{p^{\prime }-q,\gamma_{2}^{\prime }}^{+}a_{p^{\prime }\gamma_{2}}a_{p\gamma_{1}}, \end{array}$$

where *a*_**p***γ *_is the destruction operator for electron with momentum **p **from the band *γ, ξ*_*pγ *_= *γv*_F_*p*-*μ *is its kinetic energy measured from the chemical potential *μ *and *V*_*q *_= 2*πe*^2^/*εq *is the 2D fourier transform of Coulomb interaction potential screened by surrounding 3D medium with a dielectric permittivity *ε*.

## 3 Description of plasmons

To investigate the properties of plasmons in Dirac electron gas, we develop the equation-of-motion approach similarly to the original works on plasmons in usual electron gas [25]. We treat a plasmon as a composite Bose quasiparticle, consisting of electron-hole pairs with the common total momentum **q**. Thus, the creation operator for plasmon with momentum **q **can be written as:

(8) $$Q_{q}^{+}=\underset{p\gamma \gamma^{\prime }}{\sum }C_{\mathrm{pq}}^{\gamma^{\prime }\gamma }a_{p+q,\gamma^{\prime }}^{+}a_{p\gamma }.$$

Here the coefficients $C_{\mathrm{pq}}^{\gamma^{\prime }\gamma }$ are the weights of intraband (*γ *= *γ'*) and interband (*γ *≠ *γ'*) single-particle transitions, contributing to the wave function of plasmon.

The plasmon creation operator should obey Heisenberg equation of motion

(9) $$\left[H,Q_{q}^{+}\right]=\Omega_{q}Q_{q}^{+},$$

where Ω_*q *_is plasmon frequency. We start from equation of motion for single electron-hole pair, which can be derived using (7):

(10) $$\begin{array}{c} \left[H,a_{p+q,\gamma^{\prime }}^{+}a_{p\gamma }\right]\\ =\left(\xi_{p+q,\gamma^{\prime }}-\xi_{p\gamma }\right)a_{p+q,\gamma^{\prime }}^{+}a_{p\gamma }+\frac{1}{S}\underset{q^{\prime }}{\sum }V_{q^{\prime }}\rho_{q^{\prime }}^{+}\\ \times \underset{\gamma^{\prime \prime }}{\sum }\left(\left\langle f_{p+q-q^{\prime },\gamma^{\prime \prime }}|f_{p+q,\gamma^{\prime }}\right\rangle a_{p+q-q^{\prime },\gamma^{\prime \prime }}^{+}a_{p\gamma }\right)\\ \left(-\left\langle f_{p\gamma }|f_{p+q^{\prime },\gamma^{\prime \prime }}\right\rangle a_{p+q,\gamma^{\prime }}^{+}a_{p+q^{\prime },\gamma^{\prime \prime }}\right), \end{array}$$

where we have introduced the Fourier transform of the density operator:

(11) $$\rho_{q}^{+}=\underset{p\gamma \gamma^{\prime }}{\sum }\left\langle f_{p+q,\gamma^{\prime }}|f_{p\gamma }\right\rangle a_{p+q,\gamma^{\prime }}^{+}a_{p\gamma }.$$

The right-hand side of the Equation (10) contains products of four fermionic operators. To reduce it to products of two fermionic operators we use the RPA; its applicability will be discussed below. According to RPA [25], the operator products in the last two lines of (10) can be replaced by their average values in ground state |0〉 of the system:

(12) $$a_{p^{\prime }\gamma^{\prime }}^{+}a_{p\gamma }\to \left\langle 0\left|a_{p^{\prime }\gamma^{\prime }}^{+}a_{p\gamma }\right|0\right\rangle =\delta_{pp^{\prime }}\delta_{\gamma \gamma^{\prime }}n_{p\gamma },$$

where *n*_**p***γ *_is the occupation number for electronic states with momentum **p **from the band *γ*. For electron-doped Dirac liquid at *T *= 0 (see also the remark at the end of this section), we have *n*_**p**+ _= Θ(*p*_F _- |**p**|), *n*_**p**- _= 1, where *p*_F _= *μ*/*v*_F _is the Fermi momentum. In the case of hole doping, all characteristics of plasmons are the same due to electron-hole symmetry.

Thus, the equation of motion (10) for electron-hole pair with taking into account the RPA assumption (12) takes the form:

(13) $$\begin{array}{ll} & \left[H,a_{p+q,\gamma^{\prime }}^{+}a_{p\gamma }\right]=\left(\xi_{p+q,\gamma^{\prime }}-\xi_{p\gamma }\right)a_{p+q,\gamma^{\prime }}^{+}a_{p\gamma }\;\\ & \;\;+\frac{V_{q}}{S}\rho_{q}^{+}\left\langle f_{p\gamma }|f_{p+q,\gamma^{\prime }}\right\rangle \left(n_{p\gamma }-n_{p+q,\gamma^{\prime }}\right).\;\\ & \; \end{array}$$

Combining the definition of plasmon creation operator (8) with the equation of motion for plasmon (9) and single electron-hole pair (13), we obtain the system of equations for the coefficients $C_{\mathrm{pq}}^{\gamma^{\prime }\gamma }$:

(14) $$\begin{array}{ll} & \left(\Omega_{q}+\xi_{p\gamma }-\xi_{p+q,\gamma^{\prime }}\right)C_{\mathrm{pq}}^{\gamma^{\prime }\gamma }=\frac{V_{q}}{S}\left\langle f_{p+q,\gamma^{\prime }}|f_{p\gamma }\right\rangle \;\\ & \times \underset{p^{\prime }\tau \tau^{\prime }}{\sum }C_{p^{\prime }q}^{\tau^{\prime }\tau }\left\langle f_{p^{\prime }\tau }|f_{p^{\prime }+q,\tau^{\prime }}\right\rangle \left(n_{p^{\prime }\tau }-n_{p^{\prime }+q,\tau^{\prime }}\right).\;\\ & \; \end{array}$$

Introducing infinitesimal damping into the plasmon frequency and denoting

(15) $$N_{q}=\frac{V_{q}}{S}\underset{p\tau \tau^{\prime }}{\sum }C_{\mathrm{pq}}^{\tau^{\prime }\tau }\left\langle f_{p\tau }|f_{p+q,\tau^{\prime }}\right\rangle \left(n_{p\tau }-n_{p+q,\tau^{\prime }}\right),$$

we can find the plasmon wave function from (14) as:

(16) $$C_{\mathrm{pq}}^{\gamma^{\prime }\gamma }=\frac{\left\langle f_{p+q,\gamma^{\prime }}|f_{p\gamma }\right\rangle N_{q}}{\Omega_{q}+\xi_{p\gamma }-\xi_{p+q,\gamma^{\prime }}+\mathrm{i\delta}}.$$

The normalization factor *N*_**q **_can be determined from the commutation relation for plasmon operators, which should be satisfied on the average in the ground state:

(17) $$\left\langle 0\left|\left[Q_{q},Q_{q^{\prime }}^{+}\right]\right|0\right\rangle =\delta_{qq^{\prime }}.$$

Substituting (8) in (17), we get

(18) $$\underset{\gamma \gamma^{\prime }}{\sum }D_{\gamma^{\prime }\gamma }=1,$$

where the total weights of intraband (*D*_++_) and interband (*D*_+- _+ *D*_-+ _= 1 - *D*_++_) electron transitions, contributing to the plasmon wave function (16), are:

(19) $$D_{\gamma^{\prime }\gamma }=\underset{p}{\sum }{\left|C_{\mathrm{pq}}^{\gamma^{\prime }\gamma }\right|}^{2}\left(n_{p\gamma }-n_{p+q,\gamma^{\prime }}\right).$$

To find the plasmon frequency Ω_*q*_, we can substitute (16) in (15), arriving to the standard RPA equation (see it for the helical liquid [11] and for graphene [18,19]):

(20) $$1-V_{q}\Pi \left(q,\Omega_{q}\right)=0,$$

where the polarization operator of Dirac electron gas is introduced:

(21) $$\begin{array}{ll} \Pi \left(q,\omega \right) & =\frac{g}{S}\underset{p\gamma \gamma^{\prime }}{\sum }{\left|\left\langle f_{p+q,\gamma^{\prime }}\right\rangle f_{p\gamma }\right|}^{2}\;\\ & \times \frac{n_{p\gamma }-n_{p+q,\gamma^{\prime }}}{\omega +\xi_{p\gamma }-\xi_{p+q,\gamma^{\prime }}+\mathrm{i\delta}}.\;\\ & \; \end{array}$$

The degeneracy factor *g *is 1 for helical liquid on the surface of topological insulator and 4 for graphene. The angular factors 〈*f*_**p**+**q**,*γ'*_|*f*_**p***γ*_〉 are specific to helical Dirac electrons and arise in (21) as a result of summation over spinor components of electron wave function.

The RPA becomes exact in the limit of small values of dimensionless parameter *r*_s_, defined as a ratio of characteristic Coulomb interaction energy to kinetic energy. For the gas of massless particles, *r*_s _does not depend on electron density and equals to *r*_s _= *e*^2^/*εv*_F _(effectively *ε *= (*ε*_1 _+ *ε*_2_)/2 when 2D electron layer is surrounded by two dielectric half-spaces with permittivities *ε*_1 _and *ε*_2_). For Bi_2_Se_3_, *r*_s _= 0.09 with *ϵ *= 40 for dielectric half-space, and applicability of the RPA is well established (the value of *r*_s _for another material Bi_2_Te_3 _is close to that for Bi_2_Se_3_). In the case of graphene, *r*_s _is not small, but applicability of the RPA can be established due to smallness of the parameter 1/*g*, leading to selection of bubble diagrams (see [16] and references therein, and also the work [26]).

Maximal achievable amounts of doping of helical liquid on a surface of Bi_2_Se_3 _are *μ *~ 0.3 eV [24], therefore at room temperature it can be degenerate electron liquid. Hence we assume that *T *= 0 in all calculations below.

## 4 Wave function of plasmon

**Figure 1:**
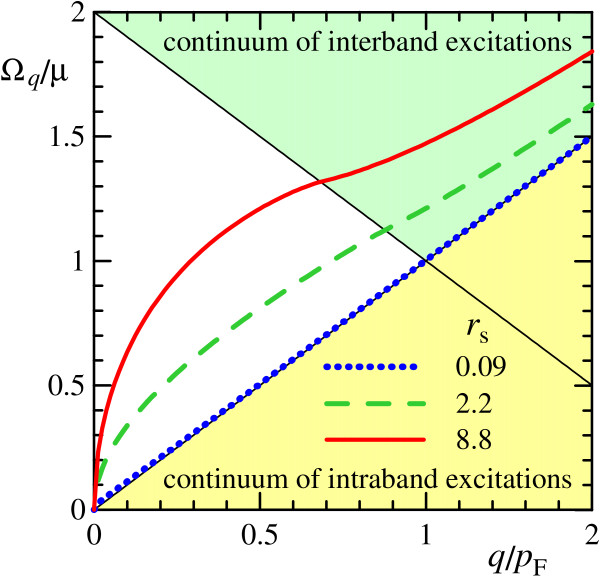
**Plasmon dispersions**. Plasmon dispersions Ω_*q *_at different values of *r*_s_. Continuums of intraband (*ω *<*v*_F_*q*) and interband (*ω *> 2*μ *- *v*_F_*q*) single-particle excitations are shaded; *p*_F _= *μ*/*v*_F _is the Fermi momentum, *μ *is the chemical potential.

Plasmon dispersions Ω_*q*_, calculated numerically from (20)-(21) at various *r*_s_, are plotted in Figure 1. We also present results for suspended graphene with *r*_s _= 8.8 (*ε *= 1) and for graphene embedded into SiO_2 _environment with *r*_s _= 2.2 (*ε *= 4). For convenience, the degeneracy factor *g *= 4 is included here into *r*_s_, since the plasmon dispersion in RPA (20) depends only on the combination *gr*_s_.

It is seen that for small values of *r*_s _(the case of topological insulator) the plasmon dispersion law approaches very closely to the upper border of the continuum of intraband single-particle transitions (*ω *<*v*_F_*q*). On the contrary, at moderate and large *r*_s_, plasmon has well-defined square-root dispersion in the long-wavelength range. At *q *≈ *p*_F_, the plasmon enters the continuum of interband single-particle transitions (*ω *> 2*μ *- *v*_F_*q*). Inside the single-particle continuum, energy and momentum conservation laws allow energy transfer between plasmon and single-particle excitations, so the plasmon acquires a finite lifetime.

**Figure 2:**
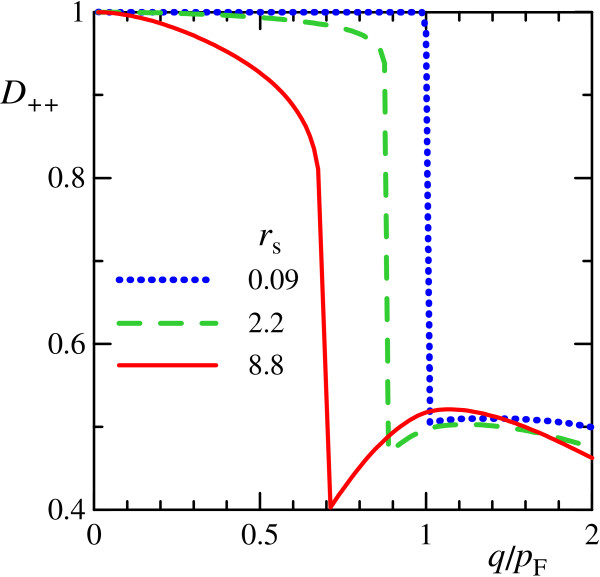
**Weight of intraband transitions in plasmon wave function**. Weight *D*_++ _(19) of intraband electron-hole transitions in plasmon wave function at different values of *r*_s_.

The internal structure of plasmons in Dirac electron gas can be characterized by total weights (19) of intra- and inter-band transitions in its wave function. The weight *D*_++ _of intraband single-particle transitions is plotted in Figure 2. It is seen that the undamped plasmon for all values of parameter *r*_s _consists mainly of intraband transitions, thus an influence of valence band on its properties is rather weak. When the plasmon enters the single-particle continuum, inter- and intra-band transitions start to contribute almost equally to its wave function, but the plasmon undergoes strong Landau damping.

**Figure 3:**
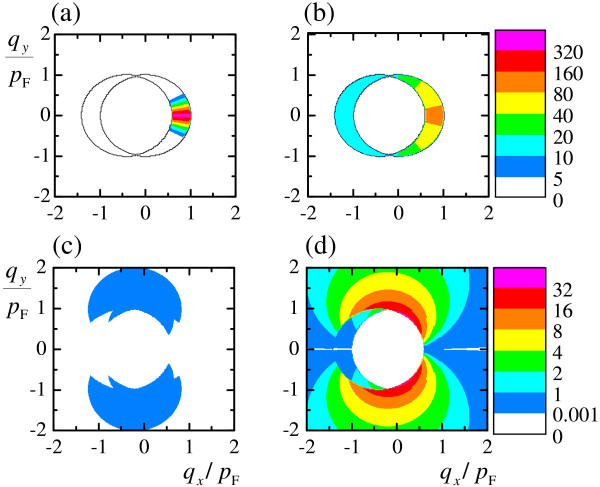
**Wave function of plasmon in momentum space**. Squared modulus of plasmon wave function ${\left|C_{\mathrm{pq}}^{\gamma^{\prime }\gamma }\right|}^{2}$ on the plane of the hole momentum **p **at *q *= 0.4*p*_F_. Top row: intraband channel ${\left|C_{\mathrm{pq}}^{++}\right|}^{2}$ at *r*_s _= 0.09 **(a) **and *r*_s _= 8.8 **(b)**. Bottom row: interband channel ${\left|C_{\mathrm{pq}}^{+-}\right|}^{2}+{\left|C_{\mathrm{pq}}^{-+}\right|}^{2}$ at *r*_s _= 0.09 **(c) **and *r*_s _= 8.8 **(d)**.

The detailed picture of the plasmon wave function in momentum space $C_{\mathrm{pq}}^{\gamma^{\prime }\gamma }$, showing the distribution of contributions of intraband ${\left|C_{\mathrm{pq}}^{++}\right|}^{2}$ or interband ${\left|C_{\mathrm{pq}}^{+-}\right|}^{2}+{\left|C_{\mathrm{pq}}^{-+}\right|}^{2}$ electron-hole pairs into wave function of plasmon with given momentum *q *is presented in Figure 3. The results are calculated for *q *= 0.4*p*_F _at *r*_s _= 0.09 and 8.8. For other values of plasmon momentum *q*, the results are qualitatively similar. It is seen that for small values of *r*_s _(the case of topological insulator) the distribution of intraband contributions is very sharply peaked in the forward direction, whereas the contribution of interband transitions is negligible. It can be understood from the reason that the plasmon dispersion is very close to the single-particle continuum (see Figure 1) and thus the plasmon itself behave almost as single intraband electron-hole transition.

At large values of parameter *r*_s _(the case of suspended graphene), the broad range of electron intraband transitions in momentum space contributes to plasmon. The weight of interband transitions is small but not negligible. Contributions of interband transitions form two side lobes, since the angular factor 〈*f*_**p**+**q**,±_|*f*_**p**∓_〉 suppresses interband forward scattering.

## 5 Charge- and spin-density waves

When a spin-plasmon is excited in the helical liquid, anisotropic distribution of electron-hole pairs of the type, depicted in Figure 3, arises. This distribution is shifted towards the plasmon momentum **q**. Due to the spin-momentum locking, the system should acquire a total nonzero spin polarization, perpendicular to **q**. A similar situation occurs in the current-carrying state of the helical liquid, which turns out to be spin-polarized [9].

Average of spin polarization in the state $|1_{q}\rangle =Q_{q}^{+}|0\rangle$ with a single plasmon excited is 〈**s**〉 = 〈1_**q**_|*σ*/2|1_**q**_〉 and can be calculated using (8) as:

(22) $$\begin{array}{ll} & \;\;\left\langle \text{s}\right\rangle =\frac{1}{2}\underset{p\gamma \gamma^{\prime }\tau }{\sum }\left[\left\langle f_{p+q,\gamma^{\prime }}\left|\sigma \right|f_{p+q,\tau }\right\rangle C_{\mathrm{pq}}^{\tau \gamma }\right)\;\\ & \left(-C_{\mathrm{pq}}^{\gamma^{\prime }\tau }\left\langle f_{p\tau }\left|\sigma \right|f_{p\gamma }\right\rangle \right]{\left(C_{\mathrm{pq}}^{\gamma^{\prime }\gamma }\right)}^{*}\left(n_{p\gamma }-n_{p+q,\gamma^{\prime }}\right).\;\\ & \; \end{array}$$

**Figure 4:**
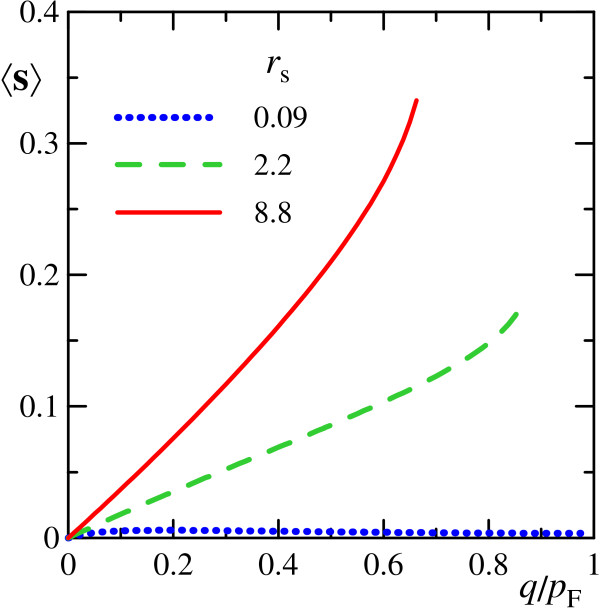
**Spin polarization of the system with an excited spin-plasmon**. In-plane transverse spin polarization of the system 〈**s**〉 (22) in the state with single spin-plasmon excited as function of its momentum *q *at different values of *r*_s_.

If **q **is parallel to **e**_*x*_, only the *y*-component of 〈**s**〉 is nonzero. Its dependence on *q *for undamped plasmons at various *r*_s _is plotted in Figure 4. At large enough *r*_s_, the spin polarization of the system is comparable with the whole spin of a single electron.

Note that in the case of graphene the isospin polarization of the system appears instead of spin polarization. Isospin polarization corresponds to nonzero average phase shift between wave function of electrons on different sublattices. On the contrary, in pseudospin-unpolarized state this shift is zero on the average.

In ordinary electronic system without spin-momentum coupling, plasmon manifests itself as charge-density wave. In helical liquid on surface of topological insulator, due to the spin-momentum locking, spin-plasmon manifests itself as coupled charge- and spin-density waves. These waves, accompanying spin-plasmon with the momentum **q**, can be characterized by corresponding spatial harmonics of charge- (11) and spin-density

(23) $$s_{q}^{+}=\frac{1}{2}\underset{p\gamma \gamma^{\prime }}{\sum }\left\langle f_{p+q,\gamma^{\prime }}\left|\sigma \right|f_{p\gamma }\right\rangle a_{p+q,\gamma^{\prime }}^{+}a_{p\gamma }$$

operators.

To relate these quantities to the plasmon operators (8), we employ the unitary transformation, connecting operators of electron-hole excitations in noninteracting system $a_{p+q,\gamma^{\prime }}^{+}a_{p\gamma }$ with operators of plasmons $Q_{q}^{+}$. For this purpose, we also need explicit expressions for operators of distorted single-particle excitations in the system with Coulomb interaction (similarly to the work [27]). Denoting creation operator for such single-particle excitation with total momentum **q **and energy $\xi_{p+q,\gamma^{\prime }}-\xi_{p\gamma }$ as $\eta_{\mathrm{pq}\gamma \gamma^{\prime }}^{+}$, we can use the equation-of-motion method in the RPA and, similarly to the case of plasmons, get the explicit expression for it:

(24) $$\eta_{\mathrm{pq}\gamma \gamma^{\prime }}^{+}=\underset{p^{\prime }\tau \tau^{\prime }}{\sum }U_{pp^{\prime }q}^{\gamma \gamma^{\prime }\tau \tau^{\prime }}a_{p^{\prime }+q,\tau^{\prime }}^{+}a_{p^{\prime }\tau },$$

where

(25) $$\begin{array}{c} U_{pp^{\prime }q}^{\gamma \gamma^{\prime }\tau \tau^{\prime }}=\delta_{pp^{\prime }}\delta_{\gamma \tau }\delta_{\gamma^{\prime }\tau^{\prime }}\\ +\frac{V_{q}}{S}\frac{\left\langle f_{p\gamma }|f_{p+q,\gamma^{\prime }}\right\rangle \left(n_{p\gamma }-n_{p+q,\gamma^{\prime }}\right)}{1-V_{q}\Pi \left(q,\xi_{p+q,\gamma^{\prime }}-\xi_{p\gamma }\right)}\\ \times \frac{\left\langle f_{p^{\prime }+q,\tau^{\prime }}|f_{p^{\prime }\tau }\right\rangle }{\xi_{p^{\prime }\tau }-\xi_{p^{\prime }+q,\tau^{\prime }}-\xi_{p\gamma }+\xi_{p+q,\gamma^{\prime }}+\mathrm{i\delta}}. \end{array}$$

The expressions (8), (16), (24), and (25) establish the unitary transformation at given **q **from operators of electron-hole excitations in noninteracting system $a_{p+q,\gamma^{\prime }}^{+}a_{p\gamma }$ to operators of excitations in Coulomb-interacting system: plasmons $Q_{q}^{+}$ and single-particle excitations $\eta_{\mathrm{pq}\gamma \gamma^{\prime }}^{+}$. We can easily derive the inverse transformation of the form:

(26) $$\begin{array}{c} a_{p+q,\gamma^{\prime }}^{+}a_{p\gamma }=\left(n_{p\gamma }-n_{p+q,\gamma^{\prime }}\right)\\ \times \left\{{\left(C_{\mathrm{pq}}^{\gamma^{\prime }\gamma }\right)}^{*}Q_{q}^{+}+{\underset{p^{\prime }\tau \tau^{\prime }}{\sum }\left(U_{p^{\prime }\mathrm{pq}}^{\tau \tau^{\prime }\gamma \gamma^{\prime }}\right)}^{*}\eta_{p^{\prime }q\tau \tau^{\prime }}^{+}\right\}. \end{array}$$

According to (26), we can represent the operators (11) and (23) of charge- and spin-density waves in the form:

(27) $$\rho_{q}^{+}=SN_{q}^{*}\Pi \left(q,\Omega_{q}\right)Q_{q}^{+}+{\tilde{\rho }}_{q}^{+},$$

(28) $${\text{s}}_{q}^{+}=SN_{q}^{*}\Pi_{s}\left(q,\Omega_{q}\right)Q_{q}^{+}+{\tilde{\text{s}}}_{q}^{+},$$

where the parts ${\tilde{\rho }}_{q}^{+}$ and ${\tilde{\text{s}}}_{q}^{+}$ are the contributions of single-particle excitations and are dynamically independent on plasmons. Here, along with the usual charge-density susceptibility (21), the crossed spin-density susceptibility of the helical liquid [11] has been introduced:

(29) $$\begin{array}{c} \Pi_{s}\left(q,\omega \right)=\frac{1}{2S}\underset{p\gamma \gamma^{\prime }}{\sum }\left\langle f_{p+q,\gamma^{\prime }}|f_{p\gamma }\right\rangle \\ \times \left\langle f_{p\gamma }\left|\sigma \right|f_{p+q,\gamma^{\prime }}\right\rangle \frac{n_{p\gamma }-n_{p+q,\gamma^{\prime }}}{\omega +\xi_{p+q,\gamma^{\prime }}+\mathrm{i\delta}}. \end{array}$$

The formulas (27)-(28) show us that the average values of $\rho_{q}^{+}$ and ${\text{s}}_{q}^{+}$ in any state with a definite number of plasmons vanish (similarly to the mean value of coordinate or momentum in the simplest quantum harmonic oscillator). However, we can calculate their mean squares in the *n*_**q**_-plasmon state $|n_{q}\rangle =[{\left(Q_{q}^{+}\right)}^{n_{q}}/{\left(n_{q}!\right)}^{-1/2}]|0\rangle$:

(30) $$\left\langle \rho_{q}\rho_{q}^{+}\right\rangle \equiv \left\langle n_{q}\left|\rho_{q}\rho_{q}^{+}\right|n_{q}\right\rangle -\left\langle 0\left|\rho_{q}\rho_{q}^{+}\right|0\right\rangle ,$$

(31) $$\left\langle s_{q}^{⊥}{\left(s_{q}^{⊥}\right)}^{+}\right\rangle \equiv \left\langle n_{q}\left|s_{q}^{⊥}{\left(s_{q}^{⊥}\right)}^{+}\right|n_{q}\right\rangle -\left\langle 0\left|s_{q}^{⊥}{\left(s_{q}^{⊥}\right)}^{+}\right|0\right\rangle$$

(only the in-plane transverse component *s*^⊥ ^of the spin s is nonzero upon averaging). Since we are interested in plasmons only, we have subtracted the vacuum fluctuations of these quantities in the ground state |0〉.

Using (27)-(28), the mean squares of charge- and spin-density wave amplitudes (30)-(31) can be easily calculated:

(32) $$\left\langle \rho_{q}\rho_{q}^{+}\right\rangle =n_{q}S^{2}{\left|N_{q}\Pi \left(q,\Omega_{q}\right)\right|}^{2},$$

(33) $$\left\langle s_{q}^{⊥}{\left(s_{q}^{⊥}\right)}^{+}\right\rangle =n_{q}S^{2}{\left|N_{q}\Pi_{s}^{⊥}\left(q,\Omega_{q}\right)\right|}^{2}.$$

**Figure 5:**
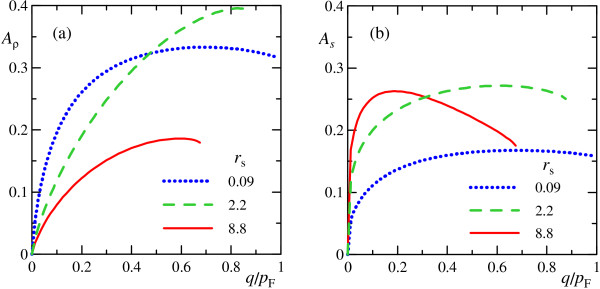
**Amplitudes of charge- and spin-density waves**. Amplitudes of charge- and spin-density waves. Normalized amplitudes of charge-density *A*_*ρ *_**(a) **and spin-density *A*_*s *_**(b) **waves in many-plasmon state as function of plasmons momentum *q *at different values of *r*_s_.

We can normalize the amplitudes to obtain dimensionless quantities $A_{\rho }\left(q\right)={\left[\left\langle \rho_{q}\rho_{q}^{+}\right\rangle /n_{q}\mathrm{S\rho}\right]}^{1/2}$ and $A_{s}\left(q\right)={\left[\left\langle s_{q}^{⊥}{\left(s_{q}^{⊥}\right)}^{+}\right\rangle /n_{q}\mathrm{S\rho}\right]}^{1/2}$ ($\rho =p_{\text{F}}^{2}/4\pi$ is the average electron density), plotted in Figure 5. As seen, the amplitudes of charge- and spin-density waves are close quantitatively at moderate momenta and any *r*_s_.

In [11], it was shown that, due to spin-momentum locking, electron density and transverse component of spin obey an analogue of "continuity equation". It requires that

(34) $$\Omega_{q}A_{\rho }\left(q\right)=2v_{\text{F}}qA_{s}\left(q\right).$$

Our results are in agreement with this equation.

## 6 Spin-plasmon scattering

Nontrivial internal structure of plasmon in Dirac electron gas can reveal itself in a process of its scattering on external potentials or impurities. For the case of spin-plasmon, it is also interesting to consider its scattering on magnetic field, acting on electron spins. Since we are interested in spin effects here, we will consider only in-plane magnetic field, affecting only spins of electrons and not their orbital motion.

Hamiltonian of interaction of electrons in helical liquid with external electric *U*(**r**) and magnetic **H**(**r**) fields are, respectively,

(35) $$H_{\text{e}}=-e\underset{pp^{\prime }\gamma \gamma^{\prime }}{\sum }U_{p^{\prime }-p}\left\langle f_{p^{\prime }\gamma^{\prime }}|f_{p\gamma }\right\rangle a_{p^{\prime }\gamma^{\prime }}^{+}a_{p\gamma },$$

(36) $$H_{\text{m}}=\mu_{\text{B}}\underset{pp^{\prime }\gamma \gamma^{\prime }}{\sum }H_{p^{\prime }-p}\left\langle f_{p^{\prime }\gamma^{\prime }}\left|\sigma \right|f_{p\gamma }\right\rangle a_{p^{\prime }\gamma^{\prime }}^{+}a_{p\gamma },$$

where *U*_**q **_and **H**_**q **_are Fourier components of external electric and magnetic fields, *μ*_**B **_= *e*/2*mc *is the Bohr magneton.

To calculate the probability of elastic scattering of the spin-plasmon with initial momentum **q **to the state with the final momentum **q***'*, we can use the Fermi golden rule, corresponding to the Born approximation. Thus, the differential (with respect to the scattering angle) probabilities of this scattering on electric and magnetic fields can be presented in the following form:

(37) $$\frac{dw_{\text{e}}}{\mathrm{d\theta}}={\left|V_{q^{\prime }-q}\right|}^{2}{\left|\Phi_{\text{e}}\left(q,\theta \right)\right|}^{2},$$

(38) $$\frac{dw_{\text{m}}}{\mathrm{d\theta}}={\left|H_{q^{\prime }-q}\cdot \Phi_{\text{m}}\left(q,\theta \right)\right|}^{2},$$

where *θ *is scattering angle (i.e., the angle between **q **and **q***'*); *q *is absolute value of both **q **and **q***'*. Here we have introduced electric Φ_e_(*q, θ*) and magnetic **Φ**_m_(*q, θ*) form-factors of spin-plasmon.

Using (35)-(36), we can calculate the form-factors explicitly:

(39) $$\begin{array}{r} \Phi_{\text{e}}(q,\theta )=e\sqrt{\frac{q}{{\Omega^{\prime }}_{q}}}\sum_{p\gamma \gamma^{\prime }\tau }(\langle f_{p+q^{\prime },\gamma^{\prime }}|f_{p+q,\tau }\rangle \\ \times C_{\mathrm{pq}}^{\tau \gamma }-C_{p+q^{\prime }-q,q}^{\gamma^{\prime }\tau }\langle f_{p+q^{\prime }-q,\tau }|f_{p\gamma }\rangle )\\ \times {\left(C_{pq^{\prime }}^{\gamma^{\prime }\gamma }\right)}^{*}(n_{p\gamma }-n_{p+q^{\prime }\gamma^{\prime }}), \end{array}$$

(40) $$\begin{array}{r} \Phi_{\text{m}}(q,\theta )=\mu_{\text{B}}\sqrt{\frac{q}{{\Omega^{\prime }}_{q}}}\sum_{p\gamma \gamma^{\prime }\tau }(\langle f_{p+q^{\prime }\gamma^{\prime }}\left|\sigma \right|f_{p+q\tau }\rangle \\ \times C_{p,q}^{\tau \gamma }-C_{p+q^{\prime }-q,q}^{\gamma^{\prime }\tau }\langle f_{p+q^{\prime }-q\tau }\left|\sigma \right|f_{p\gamma }\rangle )\\ \times {\left(C_{pq^{\prime }}^{\gamma^{\prime }\gamma }\right)}^{*}(n_{p\gamma }-n_{p+q^{\prime }\gamma^{\prime }}). \end{array}$$

Here $\Omega_{q}^{\prime }=d\Omega_{q}/\mathrm{dq}$ is the derivative of spin-plasmon dispersion law. It is convenient to project the vector **Φ**_m _on directions, parallel and perpendicular to the initial plasmon momentum **q **to get $\Phi_{\text{m}}^{∥}$ and $\Phi_{\text{m}}^{⊥}$, respectively.

**Figure 6:**
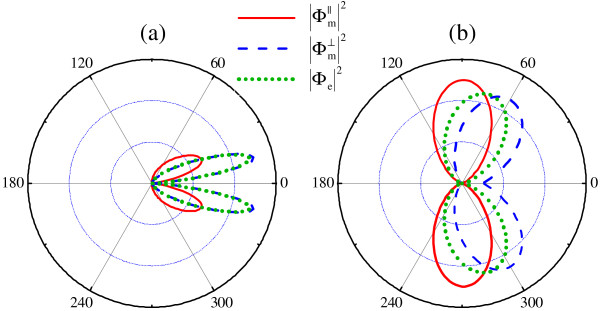
**Scattering form-factors of spin-plasmon**. Scattering form-factors of spin-plasmon. Polar graphs of the squared modulus of electric |Φ_e_(*q, θ*)|^2 ^and components ${\left|\Phi_{\text{m}}^{∥}\left(q,\theta \right)\right|}^{2}$, ${\left|\Phi_{\text{m}}^{⊥}\left(q,\theta \right)\right|}^{2}$ of magnetic form-factor of spin-plasmon with the momentum *q *= 0.6*p*_F _at *r*_s _= 0.09 **(a) **and *r*_s _= 8.8 **(b)**.

We consider only the form-factors, revealing the specifics of spin-plasmons, instead of the whole differential probabilities (37)-(38), also dependent on the form of external field. In Figure 6, angle dependencies of squared modulus |Φ_e_(*q, θ*)|^2^, ${\left|\Phi_{\text{m}}^{∥}\left(q,\theta \right)\right|}^{2}$ and ${\left|\Phi_{\text{m}}^{⊥}\left(q,\theta \right)\right|}^{2}$ of the form-factors at *q *= 0.6*p*_F _are plotted; these angular distributions are normalized to unity. In their calculation, we have used the single-band approximation, since undamped plasmons consist mainly of interband transitions, according to Figure 2. Results for other values of plasmon momentum are qualitatively the same.

In the case of forward scattering with zero momentum transfer (at *θ *= 0), the external electric field probes the total charge of plasmon, which is actually zero. Thus, the corresponding electric form-factor Φ_e_(*q*, 0) = 0. Similarly, forward scattering on magnetic field probes the total spin of spin-plasmon, which is directed perpendicularly to **q**. Therefore, $\Phi_{\text{m}}^{∥}\left(q,0\right)=0$ and $\Phi_{\text{m}}^{⊥}\left(q,0\right)\neq 0$. As for backscattering of plasmon, it is strictly prohibited, as for individual massless Dirac electrons [28].

As seen in Figure 6, the form-factors demonstrate two side lobes, rather sharp at small *r*_s_, which can be considered as a consequence of sharp peaking of the plasmon wave function (see Figure 3). At large *r*_s_, these lobes are much broader.

In the process of spin-plasmon scattering on some configurations of electric or magnetic fields, we can expect interplay between spatial structure of these fields and that of the spin-plasmon. Angular distribution of the scattered plasmons will incorporate both of these factors, according to (37)-(38). Controlling the overlap of maxima of external potential and spin-plasmon form-factors, one can manipulate spin-plasmon scattering.

Magnetic or nonmagnetic impurities can also create the external field. If the characteristic radius of the impurity potential is *R*, the transferred momentum will be limited by *R*^-1 ^by the order of magnitude. If *qR *≫ 1, the plasmon scattering on impurities will be suppressed due to proximity to the regime of forward scattering. In the opposite limit *qR *≪ 1, plasmons will be effectively scattered on considerable angles.

## 7 Conclusions

The properties of collective excitations (plasmons or spin-plasmons) in 2D gas of massless Dirac particles were studied. Two physical realizations of such systems were considered: electron gas in graphene and helical liquid on the surface of topological insulator. Quantum field-theoretical formalism for comprehensive description of spin-plasmons as composite Bose particles in the RPA was developed. Internal structure and wave function of spin-plasmons were studied.

Signatures of spin-momentum locking in helical liquid were considered. In particular, it was shown that excitation of a spin-plasmon induces the total nonzero spin polarization of the system. Moreover, coupling between charge- and spin-density waves, accompanying a spin-plasmon, was demonstrated. It was shown that amplitudes of both of these waves are close by the order of magnitude for spin-plasmons of intermediate momenta. The results of this work can be confirmed by experiments involving spin-plasmon excitation on the surface of topological insulator and independent measurements of charge and spin wave amplitudes (one of experiments of this type was proposed in [11]). The similar effect of coupling between charge and spin appears in electron gas with spin-orbit coupling [29], but amplitude of spin wave is considerable less in this case than that of charge wave for experimentally relevant parameters.

Elastic scattering of spin-plasmons in helical liquid on electric and magnetic external fields is considered. Angular distribution of scattered spin-plasmons depends on both the shape of the potential and the form-factor of the spin-plasmon, revealing its internal structure. It was shown that, due to the form-factor, the scattering occurs into two side lobes, while forward and backward scattering is suppressed. One can use this fact to manipulate spin-plasmon scattering via interplay between plasmon form-factor and shape of the external potential. It can be also concluded that scattering of spin-plasmons on long-range impurities should be very weak.

Coupling between charge and spin waves, demonstrated in this article, can be used for realization of various spintronic devices. One can perform controllable focusing of spin-plasmon waves and thus create regions with high spin polarization. Spin-polarized electrons, accumulating in these regions, can diffuse to adjacent electrodes and be used to drive spin currents (similarly to [12]).

The quantum field-theoretical approach, presented in this article, can be used for theoretical description of an influence of various external factors (impurities, external fields) on plasmons in Dirac electron gas. The obtained explicit expression for plasmon wave function allows to derive and solve Hamiltonians of plasmons interacting with such external fields. In particular, the plasmon form-factors (39)-(40) can be used to construct matrix elements of plasmon interaction with external electric and magnetic fields. The problems of 2D spin-plasmon optics, based on manipulations by inhomogeneities of the system and 3D environment, can be solved. Also the properties of hybrid modes (plasmon polaritons [30], plasmon-phonon modes [21,22], plasmon-hole modes--plasmarons [31]) can be studied using this approach.

The classical electrodynamic approach based on Maxwell equations and response functions cannot describe quantum effects, arising when individual plasmons are emitted and adsorbed. Therefore, the quantum field-theoretical approach, presented in this article, should be especially useful for the problems of quantum plasmonics, which seems to be rather feasible in graphene-based structures [23].

## Abbreviation

RPA: random phase approximation.

## Competing interests

The authors declare that they have no competing interests.

## Authors' contributions

YEL formulated the problem, provided the consultations on key points of the work and helped to finalize the manuscript. AAS developed the mathematical approach, carried out analytical calculations and contributed to the manuscript preparation. DKE performed a part of analytical calculations, obtained all numerical results and wrote the manuscript draft. All authors read and approved the final manuscript.
